# Meat and Meat Products: Explorations of Microbiota, Flavor, and Quality

**DOI:** 10.3390/foods13233900

**Published:** 2024-12-03

**Authors:** Huiping Wang, Baohua Kong, Qian Chen

**Affiliations:** College of Food Science, Northeast Agricultural University, Harbin 150030, China; whp_1998@163.com (H.W.); kongbh@163.com (B.K.)

## 1. Introduction

Meat and meat products have a very important position in the human diet, and are an important source of essential nutrients such as protein, fat, minerals, and vitamins. With the rapid pace of modern life, meat products make frequent appearances on the dinner table because of their convenience and rich taste. During the processing of meat products, raw materials such as proteins, fats, and carbohydrates undergo a series of biochemical reactions such as oxidation and hydrolysis, facilitated by microorganisms or enzymes, which results in the production of acids, alcohols, esters, and non-protein nitrogenous compounds; these give the final product its distinctive flavor [1]. Their quality characteristics, such as their texture, color, flavor, and nutritional value, are shaped by complex processes involving microorganisms, enzymes, acids, and chemical reactions, and influence consumer preferences [2].

The microbiota present in meat and meat products can have a profound impact on their quality and safety [3]. Beneficial microorganisms can contribute to preservation by producing antimicrobial substances and inhibiting spoilage and the growth of pathogenic bacteria [4]; on the other hand, harmful microorganisms can lead to spoilage and pose risks to human health [5]. Therefore, understanding the dynamics of the microbiota is crucial for developing effective preservation strategies. The microbial metabolism can produce volatile compounds that contribute to the characteristic flavor of different meat products [6]. Additionally, the interactions between microorganisms and the meat matrix can affect flavor development and stability [7]. Flavor is a key attribute that determines consumer acceptance of meat and meat products, and it is influenced by multiple complex factors, including the type of meat, the processing methods, and the presence of specific microorganisms [8]. In addition, the quality of meat and meat products is evaluated based on various parameters, such as sensory properties, nutritional value, and safety. Sensory qualities, such as color, texture, and taste, are important determinants of consumer preferences [9]. Nutritional aspects, including protein content and fatty acid composition, also contribute to overall quality [10,11]. In addition, ensuring the absence of contaminants and pathogens is essential for the safe consumption of these foods.

In recent years, numerous studies have focused on exploring the relationship between the microbiota, flavor, and quality of meat and meat products [12,13]. Advances in molecular techniques and analytical methods have provided valuable insights into the microbial communities associated with meat and their effects on flavor and quality. This Special Issue, titled “Meat Quality and Microbial Analysis II”, encompasses eight articles that delve into the microbiome and flavor of meat and meat products. In the following paragraphs, I will briefly outline these articles to encourage the reader to explore them further.

## 2. An Overview of Published Articles

The work of Wang et al. (Contribution 1) examined pathogen contamination and bacterial diversity in raw pork samples obtained from three different channels in Hangzhou. It was found that pork sold in the market demonstrated surprisingly high resistance and tolerance to chlorine, indicating the serious problem of the excessive use of antibiotics and disinfectants in pig farming in China. Thus, the detection of pathogenic bacteria in retail meat constitutes an essential element of food microbiological analysis, which can be used to detect and reduce potential health risks for consumers at an early stage. The results might have implications for public health. Therefore, the use of appropriate subsequent handling and consumption methods is necessary to guarantee food safety.

The article by Zhang et al. (Contribution 2) probed into the release of volatile compounds in mutton skewers with diverse fat-to-lean ratios. The findings indicated that the volatile components in mutton with different fat-to-lean ratios were markedly distinct. As the fat content increases, the variety and quantity of volatile substances also increase. Adding a moderate amount of fat is beneficial, enriching the volatile compounds in the oral cavity. Nevertheless, a higher fat-to-lean ratio will shorten the duration of chewing and weaken the decomposition of pellets during consumption, which is unfavorable for the potential release of volatile substances. Hence, the article set a fat-to-lean ratio of 2:2 as the best choice for making kebabs, because this can supply abundant flavor to the kebabs prior to and during consumption. The study provides a theoretical foundation for enhancing the flavor of mutton kebabs.

The article by Li et al. (Contribution 3) prepared high-activity *Lactiplantibacillus plantarum* x3-2b as a starter by adding protective agents and investigated its effects on dry fermented mutton. In the study, 15% skim milk powder and 10% trehalose were utilized as the optimal formula for freeze-drying protective agents. The prepared freeze-dried x3-2b bacterial powder could be used to improve the survival rate of the strain during the freeze-drying process. It was observed that the surface morphology of freeze-dried bacterial cells became smoother after the addition of protective agents. Moreover, the protective agent showed good encapsulation around the cells, thus reducing the damage to the cells caused by ice crystal formation. Inoculation with x3-2b bacterial powder changed the physiochemical characteristics, increased the types and numbers of flavor compounds, and decreased the biogenic amine content.

The article by Wang et al. (Contribution 4) examines the inoculation of lactic acid bacteria, along with 40% KCl, which was used to replace some NaCl. Specifically, it investigates the impacts of *L. plantarum*, *Lactobacillus sakei*, *L. curvatus*, and *Weissella hellenica* on the formation of physiochemical characteristics. The color (primarily b*-value) and texture of dry sausage can be enhanced by substituting some NaCl with KCl and inoculating with lactic acid bacteria (LAB) (especially *L. plantarum*). Additionally, the content of alcohols, acids, esters, and ketones was augmented while bitterness was reduced. A correlation analysis further validates those differences in moisture content, fat content, protein content, pH, and LAB count have significant effects on the flavor and color of dry sausages when NaCl is replaced. However, it is noted that although the correlation analysis was useful for identifying potential associations, the mechanisms through which LAB inoculation influences the flavor and quality of dry sausages require further exploration. Additionally, the optimization of LAB inoculation should be considered to further enhance the product characteristics of dry sausages. This study presents strategies and future outlooks regarding the enhancement of the quality characteristics and flavor of low-sodium dry sausages.

The article by Sui et al. (Contribution 5) also took low-sodium dry sausages as the research object. This study investigated the effect of inoculating two different yeast strains on the bacterial community structure of low-sodium dry sausages. The results demonstrate that inoculating yeast strains notably boosted the growth of bacteria and LAB and augmented the relative abundance of *Lactobacillus*, especially *L. sakei*, in low-sodium dry sausages. Consequently, the pH of the inoculated dry sausages decreased and the total acid content increased. A diversity analysis demonstrated that inoculating yeast strains had a significant impact on the bacterial composition of dry sausages. This study may help to clarify the interactions between bacterial and fungal communities in fermented dry sausages. Further investigations should center on the interaction mechanism between specific strains and fungi to supply a theoretical foundation for the artificial regulation of bacterial community functions.

The article by Liu et al. (Contribution 6) focused on dry-aged rump meat from Yanbian, China, and conducted a screening for protease-producing fungi. Four fungal strains were identified. Notably, *Yarrowia hollandica* D4 and *Penicillium oxalicum* D5 exhibited high enzyme-production activities, confirming their outstanding ability to degrade sarcoplasmic proteins, with *P. oxalicum* D5 demonstrating the greatest activity. The analysis of metabolic pathways in the fermentation broth indicated that tyrosine, tryptophan, and phenylalanine were most closely associated with the fermentation of plasma proteins via *P. oxalicum* D5. Subsequently, the selected *P. oxalicum* D5 was applied to dry-cooked beef to validate its actual effect and determine the impacts of *P. oxalicum* on protein and volatile flavor components during the fermentation of dry-aged rump meat. Ultimately, these findings present a theoretical groundwork for the advancement of starter cultures, which might expedite the development of fermented meat products.

The article by Wang et al. (Contribution 7) delved into bacterial communities. Specifically, it examined the correlation between bacterial communities and malodor formation in Xuanwei ham. The results demonstrated that microorganisms play a crucial role in the formation of ham’s (namely, Xuanwei ham’s) odor. The stink of spoiled ham is characterized by fishy, malodorous, sweaty, ichthyic, unpleasant, sour, and putrid odors, which are caused by compounds such as hexanal, trimethylamine, valeric acid, octanal, and methanethiol. These compounds showed positive and significant correlations with the presence of six strains. Additionally, a high pH, a high moisture content, the presence of thiobarbituric acid-reactive substances, and a low NaCl content are also responsible for ham deterioration. This study provides a valuable understanding of the odor of Xuanwei ham from the perspective of microbial community.

In the article by Han et al. (Contribution 8), a comprehensive analysis was conducted on the morphology, fatty acid, and metabolite characteristics of subcutaneous adipose tissue at different growth stages in Sunit sheep. The changes in adipocyte morphology, fat thickness, and fat metabolites among Sunit sheep at different growth stages were revealed. As sheep grow, fat deposition increases due to the proliferation of adipocytes. The carbon chains of fatty acids and lipids extended from Mth-6 to Mth-18 and Mth-30. Differentially expressed metabolites are mainly enriched in the key metabolic pathways, including fatty acid anabolism, glycolipid metabolism, glycolysis/gluconeogenesis, and aspartic acid metabolism. The deposition of adipose tissue might be correlated with the expression of certain metabolites, including acylcarnitines, fatty acid amides, aspartate, acetate, and phospholipids. This study uncovered the alterations in adipose tissue that occur during the growth of naturally grazed sheep and probed into the mechanism of fat metabolism. These results will contribute to future research on enhancing the quality of mutton consumption and developing the sheep industry.

## 3. Conclusions

Meat and meat products are an important part of the human diet, providing valuable nutrients and contributing to sensory enjoyment. The eight articles in this Special Issue, “Meat Quality and Microbial Analysis II”, explore the microbiota, flavor, and quality of meat and meat products from a variety of perspectives, finding that these aspects are closely intertwined. In conclusion, understanding the microbiome, flavor, and quality of meat and meat products is essential for developing innovative strategies to improve their sensory characteristics, safety, and nutritional value. Future research should focus on elucidating the complex interactions between microorganisms, flavor compounds, and quality attributes, as well as exploring new technologies and approaches to controlling the microbiome and enhancing the overall quality of these products.

## Data Availability

Data are contained within the article.
